# Emerging Role of Ubiquitin Proteasome System and Autophagy in Pediatric Demyelinating Leukodystrophies and Therapeutic Opportunity

**DOI:** 10.3390/cells13221873

**Published:** 2024-11-12

**Authors:** Dar-Shong Lin, Che-Sheng Ho

**Affiliations:** 1 Department of Translational Medicine, MacKay Memorial Hospital, 92, Section 2, Chung-Shan North Road, Taipei 10449, Taiwan; 2 Department of Pediatrics, MacKay Memorial Hospital, Taipei 10449, Taiwan; 3 Department of Medicine, MacKay Medical College, New Taipei 25245, Taiwan; 4 Department of Medical Research, MacKay Memorial Hospital, Taipei 10449, Taiwan; 5 Department of Neurology, MacKay Children’s Hospital, Taipei 10449, Taiwan; pedcsho@mmh.org.tw

**Keywords:** white matter diseases, demyelinating leukodystrophies, X-linked adrenoleukodystrophy, globoid cell leukodystrophy, autophagy, ubiquitin–proteasome system

## Abstract

Leukodystrophies represent a heterogeneous group of disorders characterized by specific genetic mutations, metabolic abnormalities, and degeneration of white matter in the central nervous system. These disorders are classified into several categories, with X-linked adrenoleukodystrophy (X-ALD), metachromatic leukodystrophy (MLD), and globoid cell leukodystrophy (GLD) being the most prevalent demyelinating leukodystrophies in pediatric populations. Maintaining proteostasis, which is critical for normal cellular function, relies fundamentally on the ubiquitin–proteasome system (UPS) and autophagy for the degradation of misfolded and damaged proteins. Compelling evidence has highlighted the critical roles of UPS and autophagy dysfunction in the pathogenesis of neurodegenerative diseases. Given the complex and poorly understood pathomechanisms underlying demyelinating leukodystrophies, coupled with the pressing need for effective therapeutic strategies, this review aims to systemically analyze the molecular and pathological evidence linking UPS and autophagy dysfunction to demyelinating leukodystrophies, specifically X-ALD and GLD. Furthermore, we will assess the therapeutic potential of autophagy modulators in the management of X-ALD and GLD, with the objective to inspire further research into therapeutic approaches that target autophagy and UPS pathways. Novel therapies that enhance autophagy and UPS function hold promise as complementary regimens in combination therapies aimed at achieving comprehensive correction of the pathogenic mechanisms in demyelinating leukodystrophies.

## 1. Introduction

Leukodystrophies are genetically inherited disorders affecting the white matter in the brain and spinal cord, while leukoencephaolpathies can be hereditary or acquired from various causes that lead to white matter damage. Epidemiological studies estimate the incidence of leukodystrophies to range between 1 in 4700 and 1 in 7633 live births, with a prevalence of 93 per 1000 in pediatric neurodegeneration cases [1,2]. Individuals affected by these conditions experience significant morbidity and are at an increased risk of early mortality.

Leukodystrophies are chiefly attributable to defects in glial cells or abnormalities in the myelin sheath, leading to pathological alterations in oligodendrocytes, myelin, astrocytes, and other non-neuronal cell types. Consequently, leukodystrophies are categorized into several distinct groups, including myelin disorders, astrocytopathies, leuko-axonopathies, microgliopathies, and leuko-vasculopathies [3]. Among these, myelin disorders are characterized by chronic deficiencies in myelin deposition, progressive myelin loss, and disruption of myelin integrity. They can be further subdivided based on their underlying pathophysiological mechanisms into three main types: hypomyelination, demyelination, and myelin vacuolization [3]. Among the various myelin disorders, several notable conditions are classified as demyelinating leukodystrophies. These include X-linked adrenoleukodystrophy (X-ALD), metachromatic leukodystrophy (MLD), globoid cell leukodystrophy (GLD), and multiple sulfatase deficiency. Although the age of onset for demyelinating leukodystrophies varies from infancy to adulthood, pediatric cases are most commonly observed in this category [2]. The diagnostic process for these conditions typically involves a combination of clinical evaluation, biochemical analysis, and genetic testing to confirm the presence of specific mutations and to distinguish between different types of leukodystrophies. Recent advances in newborn screening have significantly enhanced the diagnostic rate of these disorders, enabling earlier initiation of treatment [4,5,6]. Despite these advancements, the high morbidity and mortality associated with demyelinating leukodystrophies remain a significant challenge. This is largely due to the rapid deterioration of the disease and the current inadequacies of available treatments. To address these challenges and improve therapeutic outcomes, it is imperative to expand our understanding of the molecular and cellular mechanisms that underlie myelin disorders. This knowledge is essential for the development of targeted therapies that can effectively modify disease progression and improve quality of life for affected individuals.

The aggregation of misfolded proteins represents a critical pathological feature across a range of neurodegenerative diseases. Notable examples include the accumulation of amyloid beta peptide in Alzheimer’s disease, a-synuclein in Parkinson’s disease, superoxide dismutase in amyotrophic lateral sclerosis, huntingtin with expanded polyglutamine repeats in Huntington’s disease, and prion proteins in prion diseases [7]. The presence of these misfolded protein aggregates in neurons induces cellular toxicity by disrupting proteostasis, impairing synaptic function, and, ultimately, leading to neuronal cell death. These processes are central to the pathogenesis of neurodegenerative disorders. Additionally, the abnormal accumulation of a-synuclein has been observed in oligodendrocytes in multiple system atrophy, highlighting a broader impact on glial cells [8]. Recent research has also indicated that autophagy, a critical cellular mechanism for degrading and recycling misfolded proteins, is frequently dysregulated in demyelinating leukodystrophies [9,10,11,12]. In these disorders, the accumulation of misfolded proteins within oligodendrocytes is emerging as a significant component of the underlying disease mechanisms. This dysregulation of autophagy and the resultant protein aggregation contribute to the disruption of myelin integrity and the progression of leukodystrophic pathology.

In this review, we further assess the potential therapeutic effects of autophagy modulators in the management of X-ALD and GLD, with the aim to inspire further research into the development of therapeutic strategies targeting UPS and autophagy pathways.

## 2. Protein Degradation System: The Ubiquitin–Proteasome System and Autophagy

Cellular proteostasis is maintained by a complex network that ensures precise regulation of protein synthesis, folding, assembly, and degradation. This proteostasis network comprises various molecular chaperones and degradation systems, which play critical roles in preserving the functional integrity of the proteome [13]. Misfolded proteins are primarily degraded through two main mechanisms: macroautophagy (hereafter referred to as autophagy) and the ubiquitin–proteasome system (UPS). Dysfunction of these degradation pathways disrupts proteostasis, leading to the accumulation of toxic protein aggregates [7]. The accumulation of these misfolded proteins not only overwhelms the cellular quality control systems but also triggers a cascade of neurotoxic events that exacerbate neuronal damage and cell death.

**Figure 1:**
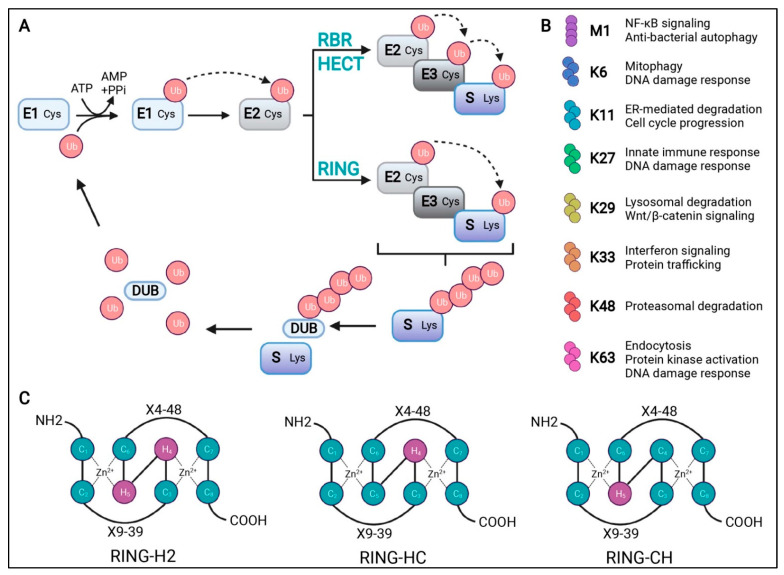
Schematic representation of the ubiquitination process. (**A**) Ubiquitination starts with an ATP-dependent step where the ubiquitin-activating enzyme (E1) creates a thioester bond between the cysteine residue at its active site and ubiquitin (Ub). Ub is then transferred to the cysteine residue in the Ub-conjugating enzyme (E2) active site. Finally, Ub ligase (E3) transfers Ub to a lysine residue of the substrate (S). There are three types of E3s, as follows: RBR, HECT, and RING domains. RBR and HECT domain E3 ligases first transfer Ub to a cysteine residue of their active site before transferring it to the substrate. The RING domain directly transfers Ub from E2 to the substrate. Deubiquitinases (DUBs) can remove, edit the length of, or disassemble a Ub chain to be recycled. (**B**) A description of the cellular function for the possible Ub chains formed through methionine (M)1 or lysine (K) 6, 11, 27, 29, 33, 48, or 63. (**C**) Schematic representation of the cross-brace arrangements of the RING-H2, RING-HC, and RING-CH finger motifs. Cysteine (C) and histidine (H) residues are numbered with their positions in the conserved zinc- (Zn^2+^-) coordination sites. X represents any amino acid located in the polypeptide between the cysteines coordinating Zn^2+^. Adopted from [15] with permission.

The UPS is the major protein degradation system responsible for nearly 80% of protein turnover [13]. This system is crucial for maintaining cellular homeostasis by regulating the degradation of proteins through a highly coordinated, ATP-dependent process known as ubiquitination [13,14]. The components of UPS include ubiquitin, ubiquitin-activating enzymes (E1), ubiquitin-conjugating enzymes (E2), ubiquitin ligases (E3), and proteasome, which consists of 20S core particles and 19S regulatory particles. The ubiquitination pathway initiates with the activation of ubiquitin by E1 enzymes (Figure 1) [15]. In this initial phase, ubiquitin is covalently conjugated to E1 via a high-energy thioester bond, a process that is energetically supported by ATP hydrolysis. Once ubiquitin is activated, it is subsequently transferred to E2 enzymes. These E2 enzymes work in concert with E3 ligases to facilitate the attachment of ubiquitin to specific lysine residues on target proteins [13,14]. This conjugation event results in the formation of a polyubiquitin chain, which acts as a molecular signal for the proteasomal degradation of the tagged protein. Proteins that have been tagged with polyubiquitin chains are specifically recognized by the 26S proteasome, a substantial proteolytic complex composed of a 20S catalytic core and one or two 19S regulatory particles [14,16]. The 19S regulatory particles are integral to several key processes involving in the recognition of substrates primarily through lysine 48 (K48), K63, and K11 linkages, the removal of ubiquitin chains (deubiquitination), and the unfolding of the target protein [17,18]. These actions are essential for facilitating the translocation of the substrate into the 20S core of the proteasome. Within the 20S core, the protein is degraded into smaller peptides and amino acids, which are then available for recycling and incorporation into new proteins [19]. This process is critical for maintaining cellular homeostasis and regulating numerous physiological functions. In the nervous system, UPS plays a critical role in elimination of damaged and misfolded intracellular protein, thereby preventing the accumulation of toxic proteins and facilitating protein recycling. However, in the context of neurodegenerative diseases, such as Alzheimer’s disease, Parkinson’s disease, and Huntington’s disease, the accumulation of misfolded proteins can overwhelm the UPS [20]. This overload may either inhibit proteasome activity or sequester the proteasome components, leading to a significant impairment in proteasomal degradation processes. The inability of neurons to effectively eliminate these aggregates and misfolded proteins can trigger cellular apoptosis, neuroinflammation, and ultimately cell death. For instance, studies have shown that mice with motor-neuron-specific disruptions in the proteasome subunit Rpt3 exhibit the accumulation of proteins linked to ALS, resulting in progressive loss of motor neurons and gliosis [21]. Additionally, individuals harboring mutations in ubiquitin protein ligases such as UBE3A, UBE3B, and ARIH2 are associated with neurodevelopmental disorders [22,23,24]. These findings underscore the critical role of UPS functionality in maintaining neuronal health and highlight its implications in various neurodegenerative conditions.

**Figure 2:**
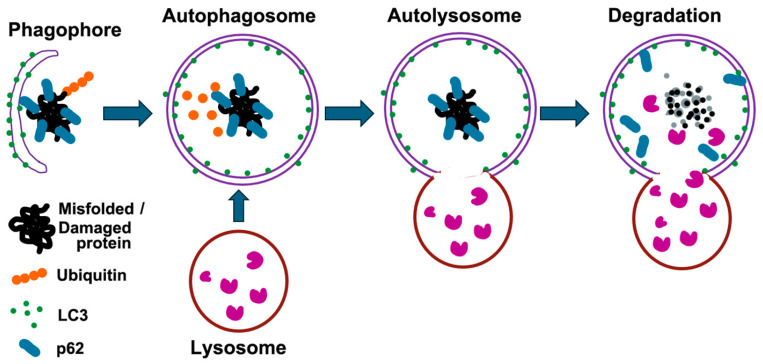
Schematic representation of the autophagy process. Autophagy is a cellular process that degrades misfolded proteins and damaged organelles. It begins with the formation of a phagophore that engulfs these components, aided by LC3, which converts to its lipidated form (LC3-II) and integrates into the autophagosomal membrane. p62 acts as a receptor for ubiquitinated substrates, linking them to LC3. The autophagosome then fuses with a lysosome to form an autolysosome, where lysosomal enzymes degrade the contents. This process recycles amino acids and other metabolites, maintaining cellular homeostasis and preventing the accumulation of damaged proteins.

The UPS primarily degrades short-lived, misfolded, and damaged proteins, accounting for approximately 80% of cellular protein turnover. In contrast, autophagy serves as a complementary pathway that targets larger misfolded proteins and damaged organelles that cannot be efficiently processed by the proteasome due to their size and structural complexity [13,25]. The autophagy adaptor p62/SQSMT1 (hereafter p62) interacts with ubiquitin noncovalently via their ubiquitin-binding domains and then delivers polyubiquitinated cargoes to autophagy [26]. Upon the initiation of autophagy, a crescent-shaped double membrane, which is closely associated with microtubule-associated protein 1 light chain 3 (LC3), engulfs the p62-mediated polyubiquitinated cargoes to form autophagosomes (Figure 2) [25]. Subsequently, the autophagosome fuses with lysosomes, resulting in the formation of autolysosomes. Within these autolysosomes, the ubiquitinated cargoes, along with the LC3 and p62 adaptors, are degraded by lysosomal hydrolases.

Recent studies have highlighted the interplay between the UPS and autophagy, suggesting that these two pathways are not entirely independent but, rather, functionally interconnected [27]. For instance, the accumulation of misfolded proteins can overwhelm the UPS, leading to increased reliance on autophagy for the clearance of aggregates. Conversely, autophagy can also regulate the availability of ubiquitin and other components necessary for UPS function, thereby maintaining overall proteostasis.

## 3. Dysfunction of Autophagy and UPS in X-ALD

X-linked adrenoleukodystrophy (X-ALD) represents the most prevalent peroxisomal disorder, attributable to mutations in the ABCD1 gene located on the X chromosome at position Xq28. This gene encodes the ATP-binding cassette, subfamily D, member 1 (ABCD1) transporter, which is located in the peroxisomal membrane. The structure of human ABCD1 reveals a dimeric assembly composed of two identical subunits. Each subunit is composed with transmembrane domains (TMDs) facilitating the transport of VLCFAs, nucleotide-binding domains (NBDs) for binding and hydrolyzing ATP, and c-terminal coiled-coil domains modulating ATPase activity [28,29]. The ABCD1 transporter is crucial for the importation of very-long-chain fatty acids (VLCFAs) and VLCFA-CoA esters into the peroxisome [30]. Within the peroxisome, these substrates are subjected to degradation via beta-oxidation. Dysfunction in the ABCD1 transporter impedes this process, leading to the accumulation of VLCFAs in various tissues and bodily fluids. Diagnostic assessment typically includes anamnesis, magnetic resonance imaging (MRI), and analysis of VLCFA profiles, specifically measuring elevated levels of hexacosanoic acid (C26:0), the ratio of C24:0 to C22:0, and the ratio of C26:0 to C22:0 [30]. X-ALD presents with three primary phenotypic forms: cerebral childhood ALD (cALD), adult-onset adrenomyeloneuropathy, and primary adrenal insufficiency [4]. Cerebral childhood ALD, the most severe form, predominantly affects males between the ages of 5 and 12. This phenotype is characterized by progressive behavioral, cognitive, and neurological impairments, often culminating in death within a few years of diagnosis. Despite the identification of over 2700 variants of the ABCD1 gene to date, there remains no definitive correlation between specific genotypes and phenotypic outcomes [31]. This highlights the complexity of the disease and underscores the need for further research to elucidate the mechanisms driving phenotypic variability.

The contribution of VLCFA-induced oxidative stress to the pathogenesis of ALD has been increasingly recognized. Both in vivo and in vitro studies demonstrate that oxidative stress induced by hexacosanoic acid (C26:0) results in the accumulation of polyubiquitinated conjugates. This process is associated with an upregulation of proteasome components and concurrent inhibition of proteasome function [32].

Research indicates that oxidative modification of proteins adversely affects the UPS, a crucial mechanism for protein degradation. Alongside UPS dysfunction, there is substantial evidence of autophagy impairment in ALD [32]. Specifically, studies have reported decreased levels of LC3-II and increased levels of p62 in the brain tissue of X-ALD patients as well as in the spinal cords of murine models, suggesting a disruption in autophagic flux [10]. Further validation comes from observations in X-ALD patient fibroblasts, which show a reduced formation of autophagosomes, accompanied by lower LC3-II levels and higher p62 levels. These findings indicate a compromised autophagic process. Moreover, X-ALD fibroblasts exhibit significantly diminished total degradation of long-lived proteins and reduced lysosomal degradation capacity. Collectively, these findings, in conjunction with prior research, underscore that the accumulation of VLCFAs impairs both the UPS and autophagy [10,32]. These disruptions are likely major contributors to the axonal degeneration observed in X-ALD, highlighting the complex interplay between oxidative stress, protein degradation pathways, and neurodegenerative processes in the disease.

Consequently, the therapeutic potential of pharmacological interventions aimed at enhancing autophagy has been explored in models of X-ALD. Research has demonstrated that temsirolimus, an ester of rapamycin and an inhibitor of the mechanistic target of rapamycin complex-1 (mTORC1), can effectively restore autophagic activity. This treatment alleviates the accumulation of oxidized proteins and mitigates proteasome dysfunction in the spinal cords of X-ALD mice [10]. Notably, temsirolimus also reduces the activation of reactive astrocytes and microglia, which are indicative of neuroinflammation. Furthermore, it prevents axonal damage and impedes the progression of locomotor deficits in these animal models [10]. These findings collectively suggest that pharmacological strategies targeting autophagy, such as mTOR inhibition with temsirolimus, hold promise as potential therapeutic approaches for X-ALD. They highlight the efficacy of modulating autophagic pathways to counteract some of the neuropathological features associated with the disease.

## 4. Dysfunction of Autophagy and UPS in GLD

GLD, commonly referred to as Krabbe disease, is an autosomal lysosomal storage disease caused by deficiency of lysosomal enzyme galactocerebrosidase (GALC), which catabolizes the degradation of galactosyl-ceramide in the initial step of myelination synthesis and the degradation of toxic galactosyl-sphingosine (psychosine) [33,34]. The deficiency of GALC activity leads to profound accumulation of psychosine impairing the myelin synthesis and deposition resulting in the widespread demyelination in both central (CNS) and peripheral nervous systems (PNS) [35]. Symptom onset of GLD varies from early infancy to adulthood, when the progression of demyelination reaches a level to impair the integrity of neuronal function. The infantile form comprises nearly 90% of GLD cases and presents the most severe phenotype, characterized by high morbidity and mortality [36,37]. The symptoms commence with restless, hypotonia, and irritability as early as 2 months of age, followed by seizures, tonic spasms, clonus, weakness, feeding difficulty, and failure to thrive. Affected infants rapidly regress to a state of severe incapacitation and often succumb to the disease by the age of two.

Research into the pathophysiology and potential interventions for GLD has been significantly advanced with the twitcher mouse model. These mice, which naturally harbor mutations in the *galc* gene, exhibit an accumulation of psychosine, extensive neuroinflammation, widespread demyelination in both CNS and PNS, and succumb to death around 40 days of age [38]. This model closely mirrors the human condition, providing valuable insights into the disease mechanisms and potential therapeutic approaches

Several reports have indicated the critical role of toxic psychosine contributing to the rapid loss of oligodendrocytes and the neurological deterioration, leading to the proposal the “psychosine hypothesis” underlying the pathogenesis of GLD [39,40]. This hypothesis was recently validated by the elimination of psychosine accumulation through the ablation of acid ceramidase in GALC-deficient twitcher mice, leading to the rescue of demyelination in cerebellar white matter and sciatic nerves [41]. In in vitro studies, endogenous psychosine accumulation in oligodendrocytes under GALC deficiency impedes the synthesis of myeline constituents and induces apoptotic cell death of maturing oligodendrocytes [42]. Consistent with this finding, oligodendrocytes of twitcher mice exhibit intrinsic impairment of differentiation, myelin formation, and cellular survival associated with the aberrant accumulation of endogenous psychosine in vivo and in vitro [35]. Research conducted by Castelvetri et al. further indicated that elevated levels of psychosine block antegrade and retrograde fast axonal transport through exacerbation of the GSK3β pathway triggering dying-back degeneration and demyelination, while inhibition of GSK3β pathway significantly ameliorates defects of fast axonal transport in vivo and in vitro [43]. These findings provide compelling evidence that the endogenous accumulation of psychosine impairs the differentiation, maturation, and survival of oligodendrocytes. Furthermore, psychosine accumulation initiates axonal dying-back degeneration and increases neuronal vulnerability by obstructing anterograde and retrograde fast axonal transport. Despite these insights, the precise pathomechanism of demyelination in GLD remains elusive, necessitating further investigation to fully elucidate the underlying processes.

Recent studies have observed autophagy dysfunction in both in vivo and in vitro models using twitcher mice and oligodendrocytes derived from human and twitcher mice. Notably, oligodendrocytes from the cortex of twitcher mice exhibit increased levels of cleaved LC3B colocalized with lysosomes following psychosine treatment [44]. By immunohistochemistry and Western blotting analysis, p62 expression and aggregates were markedly increased in brains and sciatic nerves of twitcher mice in both early and late symptomatic stages in comparison with those of wild-type mice [12]. These findings suggest a critical involvement of autophagy in the molecular pathogenesis of GLD.

Further investigations have demonstrated that psychosine-induced impairment of autophagy and the UPS underlies the demyelination observed in GLD [11]. Specifically, levels of cleaved LC3B, p62, and ubiquitin in the insoluble phase are markedly increased in the brainstem of twitcher mice at late symptomatic stages compared to wild-type mice, with insoluble p62 coprecipitating with insoluble ubiquitin. In contrast, the levels of soluble p62 and ubiquitin remain indistinguishable between twitcher and wild-type mice.

Using immunohistochemistry staining at various postnatal days, we first documented the spatiotemporal accumulation and distribution of ubiquitin and p62 aggregates during disease progression in twitcher mice brains. These aggregates are primarily detected in the white matter at presymptomatic stages and become more pronounced as the disease progresses. Intriguingly, the distribution of ubiquitin and p62 aggregates follows a caudal-to-rostral axis, extending from the spinal cord, brainstem, and cerebellar white matter to the corpus callosum. This pattern aligns with the deterioration of demyelination and the worsening phenotype, suggesting a close relationship between impaired degradation of misfolded proteins and GLD pathogenesis [11]. Double-staining and immunofluorescent analyses have validated the colocalization of ubiquitin with p62 in aggregates, which predominantly deposit in the cytoplasm of oligodendrocytes and among corrupted fiber bundles of white matter. Astrocytes and microglia are devoid of these aggregates, and neurons with aggregates are sparsely found in the brainstem, spinal cord, and granular cells of the cerebellum at late stages. These findings indicate that disturbed proteostasis leads to the deposition of insoluble misfolded proteins into aggregates within the cytoplasm of oligodendrocytes, resulting in cell death and subsequent global demyelination in GLD.

In vitro studies have demonstrated that levels of insoluble ubiquitin and p62 are markedly elevated in human oligodendrocytes under psychosine treatment, with a significant increase observed following the inhibition of autophagy. Consistent with these findings, the inhibition of the UPS under psychosine conditions significantly elevates the levels of insoluble ubiquitin and p62, an effect that is further amplified when both autophagy and UPS are inhibited simultaneously [11]. Intriguingly, psychosine treatment induces the cytoplasmic deposition of ubiquitin and p62 aggregates in oligodendrocytes. This deposition becomes more pronounced with the inhibition of either autophagy or UPS alone and results in large, pleomorphic aggregates when both pathways are inhibited concurrently. The accumulation of these aggregates in the cytoplasm of oligodendrocytes leads to increased oxidative stress, impaired bioenergetic function, and reduced cell viability [11]. These findings highlight the susceptibility of oligodendrocytes to psychosine-induced toxicity, particularly regarding the handling of misfolded and damaged proteins. The accumulation of such aggregates is believed to play a significant role in the pathogenesis of GLD. The interplay between impaired protein degradation pathways, specifically autophagy and UPS, exacerbates the cellular stress response, contributing to the neurodegenerative processes observed in GLD. Furthermore, understanding the molecular mechanisms underlying the aggregation of ubiquitin and p62 in the context of psychosine exposure could provide insights into novel therapeutic targets. By focusing on enhancing the efficiency of autophagy and UPS, it may be possible to develop interventions that not only alleviate the symptoms of GLD but also address the underlying pathogenic processes.

## 5. Potential Therapeutic Approaches for GLD

To date, there is no definitive therapeutic cure for GLD. Hematopoietic stem cell transplantation (HSCT) administered within the first two months of life, prior to the onset of symptoms, has been shown to improve survival rates and functional abilities in infants with GLD. However, long-term follow-up indicates that surviving patients continue to experience developmental delays and motor disabilities [45,46,47]. For the majority of GLD patients, current treatments are primarily limited to symptomatic and supportive care. These outcomes underscore the urgent need for the development of more effective therapeutic strategies for GLD. Attempts to directly administer the recombinant GALC enzyme in twitcher mice have not succeeded in ameliorating neuropathology or extending lifespan [48]. In contrast, bone marrow transplantation (BMT) has demonstrated efficacy by increasing GALC enzyme levels, reducing psychosine accumulation, mitigating inflammation and demyelination, and extending the lifespan of twitcher mice to approximately 80 days [49]. Moreover, CNS-directed gene therapy has emerged as a promising approach, delivering rapid, widespread, and stable GALC activity within the CNS of twitcher mice. This method has resulted in the amelioration of pathological features and an extension of lifespan by 2–3 weeks [50,51,52,53]. Notably, the combination of CNS-directed gene therapy and BMT has shown synergistic efficacy, significantly improving demyelination in both the CNS and PNS and extending lifespan by 8 to 30 weeks. Despite these advancements, the median lifespan of treated mice remains shorter than that of wild-type mice, and long-term survivors exhibit late-onset pathological phenotypes [54,55,56]. These findings suggest a conceptual framework for further research into additional pathogenic mechanisms driven by psychosine. They also highlight the potential for therapeutic interventions that target secondary disease mechanisms as complementary strategies to current treatments.

Given the role of autophagy dysfunction in the pathogenesis of GLD, pharmacotherapy targeting autophagy represents a potential strategy for modifying the disease process. Lithium, an autophagy modulator commonly used in the treatment of bipolar disorder, has demonstrated efficacy in preventing alpha-synuclein-mediated protein accumulation and aggregation, as well as protecting neurons from oxidative-stress-induced cell death in models of Parkinson’s disease [57].

Recently, the therapeutic potential of lithium in GLD was investigated using the twitcher mouse model [58]. Chronic administration of lithium, initiated at the presymptomatic stage, was found to have no significant impact on the autophagy pathway, myelination, gliosis, or neurobehavioral performance in these mice [58]. Of note, lithium exerts its effects by activating autophagosome-mediated proteolysis through the inhibition of inositol monophosphatase, leading to a reduction in free inositol and myo-inositol-1,4,5-triphosphate levels [59]. These findings suggest that further optimization of lithium administration protocols and dosages, or the selection of alternative autophagy modulators such as rapamycin derivatives, may be necessary to effectively activate autophagy in GLD.

In our previous studies, rapamycin has been shown to significantly impact the accumulation of p62 and ubiquitin aggregates in the brains of twitcher mice [60]. Research indicates that the administration of rapamycin not only reduces the deposition of insoluble ubiquitin and p62 aggregates but also mitigates the overall burden of protein aggregates associated with the disease. In twitcher mice, rapamycin treatment leads to a marked reduction in the levels of insoluble ubiquitin aggregates compared to untreated mice. Specifically, the total number of ubiquitin aggregates is reduced by nearly 50%, and the area occupied by these aggregates is significantly diminished. Furthermore, the average size of these aggregates in regions such as the corpus callosum and cerebellum is also decreased following rapamycin treatment. However, it is important to note that while rapamycin alleviates the accumulation of insoluble ubiquitin, it does not significantly alter the overall levels of soluble p62 when compared to untreated twitcher and wild-type mice. The mechanism underlying these effects is linked to rapamycin’s role as an mTOR inhibitor. By inhibiting mTOR signaling in the brain, rapamycin enhances autophagic flux and the UPS, both of which are crucial for the degradation of misfolded and aggregated proteins. In twitcher mice, the dysfunction of these pathways leads to the accumulation of insoluble p62 and ubiquitin, contributing to the neurodegenerative pathology observed in GLD. The treatment with rapamycin appears to restore some degree of function to these pathways, thereby facilitating the clearance of protein aggregates. The findings regarding rapamycin’s effects on p62 and ubiquitin aggregates underscore the potential of targeting autophagy and the UPS as therapeutic strategies for GLD. By enhancing the degradation of protein aggregates, rapamycin not only alleviates the pathological features of the disease but also improves neuroinflammation, demyelination, and overall disease progression. This suggests that rapamycin and similar compounds could represent valuable components of a multifaceted therapeutic approach aimed at managing GLD and potentially other neurodegenerative disorders characterized by protein aggregation. However, it is critical to address the challenges associated with the administration and delivery of autophagy modulators, such as rapamycin derivatives and lithium, particularly regarding their ability to cross the blood–brain barrier and the adverse effects posed by chronic administration.

## 6. Conclusions

Demyelinating leukodystrophies represent a significant challenge in pediatric neurodegenerative disorders. This review article aims to provide a comprehensive overview of recent advancements in research concerning the UPS and autophagy in pediatric leukodystrophies, highlighting their potential for novel therapeutic strategies. Compelling evidence indicates that dysfunctions in UPS and autophagy play critical roles in the pathogenesis of X-ALD and GLD. Consequently, pharmacotherapy targeting UPS and autophagy has emerged as a potential therapeutic strategy for modifying the disease processes in these demyelinating leukodystrophies. However, given the limited therapeutic efficacy of current approaches, there is a growing recognition that combining different therapeutic regimens may enhance efficacy by addressing various aspects of the pathogenic deficits.

This review article further seeks to inspire the optimization of therapeutics aimed at targeting UPS and autophagy. Future research should prioritize the development and refinement of these combination therapies to maximize their therapeutic efficacy and improve clinical outcomes for patients afflicted with demyelinating leukodystrophies.

## Data Availability

Not applicable.
